# Reassessment of asymptomatic carriers of *Plasmodium* spp. in an endemic area with a very low incidence of malaria in extra-Amazonian Brazil

**DOI:** 10.1186/s12936-017-2103-6

**Published:** 2017-11-09

**Authors:** Filomena E. C. de Alencar, Rosely dos Santos Malafronte, Crispim Cerutti, Lícia Natal Fernandes, Julyana Cerqueira Buery, Blima Fux, Helder Ricas Rezende, Angelica Espinosa Miranda

**Affiliations:** 1 Graduate Programme in Infectious Diseases, Federal University of Espírito Santo, Vitória, Brazil; 2 Protozoology Laboratory, Institute of Tropical Medicine, University of São Paulo, São Paulo, Brazil; 3 Entomology and Malacology Unit, Espírito Santo State Department of Health (SESA), Vitória, Brazil

**Keywords:** Malaria, *Plasmodium vivax*, *Plasmodium malariae*, Carrier state, Polymerase chain reaction

## Abstract

**Background:**

Regions with residual transmission are potential obstacles to the elimination of malaria. It is, therefore, essential to understand the factors associated with the maintenance of endemic malaria in these areas. The objective was to investigate whether the status of asymptomatic carriers of *Plasmodium* spp. DNA is maintained in the long term in an extra-Amazonian region of Brazil with low incidence, residual malaria transmission.

**Methods:**

Asymptomatic carriers of *Plasmodium* DNA detected in a survey carried out between 2001 and 2004 were reassessed between 2010 and 2011 using questionnaires, PCR and thick and thin blood smear tests three times at 3-month intervals.

**Results:**

Of the 48 carriers detected between 2001 and 2004, 37 were located. Of these, only two had positive PCR results and, as in the first survey, *Plasmodium malariae* DNA was detected.

**Conclusion:**

The findings suggest that untreated dwellers from this extra-Amazonian region, who initially harbour malaria parasites, may become negative without ever developing apparent symptoms of the disease. Although the possibility of re-infection cannot be ruled out, the finding of two individuals harbouring *P. malariae*, both in the first and in the second survey, may be compatible with a long-term carrier state for this parasite. Since most clinical cases of malaria in the region are a consequence of infection by *Plasmodium vivax*, the epidemiological impact of such long-term carriage would be limited.

## Background

Although malaria control efforts have reduced the impact of the disease around the world, there were an estimated 212 million cases worldwide in 2015, leading to 429,000 deaths [1]. Despite the reduced incidence of the disease in most transmission areas as a result of malaria control strategies, the existence of residual transmission in some regions, such as parts of the Atlantic Forest in extra-Amazonian Brazil, can make it difficult to achieve the goal of worldwide elimination of the disease.

Residual malaria in the Atlantic Forest in Brazil has a very low incidence in various states along the coast. The greatest number of reported cases in these states is in Espírito Santo [2], where the disease occurs in the mountainous region, which lies no more than 50 km on average from the coast. Seventeen to 68 cases are reported every year in an area covering approximately 5343 sq km [3]. In a survey carried out in this area between 2001 and 2004 [4], *Plasmodium vivax* was identified in 48 of the 51 symptomatic individuals previously diagnosed as malaria cases by both microscopic examination of blood films and polymerase chain reaction (PCR) who were asked to participate in the survey. *Plasmodium malariae* DNA was identified by PCR in one sample, and in two samples blood smear tests revealed parasites morphologically similar to *P. vivax* but the PCR was negative. At the same time, 1527 blood samples were collected from residents of the areas where cases were reported and tested by multiplex PCR and microscopic analysis of blood films (thick and thin smears) to identify asymptomatic carriers. *Plasmodium vivax* DNA was detected by PCR in 23 of the samples, *P. malariae* DNA in 15, *Plasmodium falciparum* DNA in nine, and *P. falciparum* and *P. malariae* DNA in one. No parasites were observed in the thick smears of these asymptomatic carriers.

Understanding the transmission chain associated with endemic malaria maintained in such a vast area with such a low frequency of symptomatic and asymptomatic cases is a considerable challenge. According to one hypothesis, this behaviour may be typical of a zoonosis and the human cases may be a result of incidental transmission of locally occurring simian malaria [5–7], which in Brazil is caused by two *Plasmodium* species, *Plasmodium brasilianum* and *Plasmodium simium*. The genetic similarity between *P. vivax* and *P. simium* and between *P. malariae* and *P. brasilianum* [8, 9] reinforces the possibility of a zoonosis, with simians acting as reservoirs in the transmission cycle involving humans. However, despite the low frequency of the cases, it is important to determine whether the low-endemic malaria could be maintained by asymptomatic human carriers. The aim of this study was therefore to reassess, clinically and parasitologically, the individuals who in the first survey tested positive for the presence of *Plasmodium* DNA in the bloodstream. Consequently, this reassessment enables the verification of long-term positivity for *Plasmodium* DNA and clinical evolution to apparent disease, contributing to the understanding of the role of asymptomatic carriers in the sustained transmission of malaria among humans in this endemic area.

## Methods

### Design

Of 48 asymptomatic individuals who were positive for *Plasmodium* DNA in the first survey (between 2001 and 2004), 37 were located in 2010 and included in the study. The remaining 11 consisted of three who had died of causes unrelated to malaria (stroke, acute myocardial infarction and respiratory insufficiency secondary to chronic obstructive pulmonary disease), one who refused to take part in the study and seven who had moved and could not be located. Each of the individuals located was asked to provide a blood sample once every 3 months on three separate occasions to investigate the presence of *Plasmodium* DNA. Before the first collection the participants signed a voluntary informed-consent form. On each occasion a questionnaire was filled out covering demographic data and any instances since the previous collection of febrile illnesses, thick-smear tests for malaria or trips outside the area where the participant lived. A total of 5 mL of blood was then collected in vacutainer tubes (BD Vacutainer™, New Jersey USA) containing EDTA, thick and thin peripheral blood smears were prepared and the subject’s spleen was palpated by the physicians in charge of the project (FECA and CCJ).

The blood collected was centrifuged at 300*g* for 10 min in the Protozoology Laboratory in the Tropical Medicine Unit, Federal University of Espírito Santo, to separate the plasma from the red blood cells. These were then aliquoted and stored at − 20 °C in Eppendorf tubes, which were sent to the Protozoology Laboratory at the São Paulo Institute of Tropical Medicine, where DNA was extracted and PCR was performed.

### Thick and thin smears

Thick and thin blood smears were prepared following the method recommended by the World Health Organization [10]. The smears were examined under an optical microscope at 1000 × magnification in the malaria sector of the Central Public Health Laboratory of the State of Espírito Santo by experienced senior microscopists. The results were based on examination of at least 200 microscopic fields.

### Amplification of *Plasmodium* DNA

Genomic DNA was extracted from the samples with the NucleoSpin Tissue purification kit (Macherey–Nagel) following the manufacturer’s instructions. The PCR procedure followed Win et al. [11]. Briefly, nested-PCR, which consists of two rounds of amplification, was carried out with primers that target the *Plasmodium* 18S RNA gene sub-unit. The first step was carried out in a final volume of 20 µL containing 0.8 µL of each primer, P1UP and P2 (10 µM), 0.25 µL of dNTP mix (10 mM each, Thermo Fisher Scientific), 2 µL of 10 × PCR buffer, 1 µL of MgCl_2_ (50 mM), 0.16 µL of Platinum Taq DNA polymerase-Invitrogen (5 U/µL) and 5 µL of DNA. The amplification was run in an Applied Biosystems thermocycler with the following programme: 92 °C for 2 min, 35 cycles of 92 °C for 30 s and 60 °C for 90 s and one cycle of 60 °C for 5 min.

The product from the first step was diluted 1:50 in sterile water and used in the second step with the P1 (genus–specific) primer and one of the three reverse species-specific primers (V1—*P. vivax*, F2—*P. falciparum* or M1—*P. malariae*).

This step was carried out in a final volume of 20 µL containing 2 µL of each primer (10 µM), 0.5 µL of dNTP (10 mM each, Thermo Fisher Scientific), 2 µL of 10 × PCR buffer, 0.16 µL of Taq DNA polymerase (5 U/µL) and 2 µL of the diluted final product from the first step. The second amplification was carried out in the same equipment as the first, and the cycling parameters were 92 °C for 2 min followed by 18 cycles of 92 °C for 30 s and 60 °C for 1 min and one cycle of 60 °C for 5 min. The amplified products of the second step correspond to species-specific fragments of about 100 bp.

The primers used were:

P1UP (F): 5′ TCC ATT AAT CAA GAA CGA AAG TTA AG 3′
P2 (R): 5′ GAA CCC AAA GAC TTT GAT TTC TCA T 3′
P1 (F): 5′ ACG ATC AGA TAC CGT CGT AAT CTT 3′
V1 (R): 5′ CAA TCT AAG AAT AAA CTC CGA AGA GAA A 3′
F2 (R): 5′ CAA TCT AAA AGT CAC CTC GAA AGA TG 3′
M1 (R): 5′ GGA AGC TAT CTA AAA GAA ACA CTC ATA T 3′.

DNA extracted from peripheral blood of *P. vivax* malaria patients treated at the São Paulo Department for the Control of Endemic Diseases (SUCEN), from *P. falciparum* cultures and from blood smears positive for *P. malariae* provided by the Centers for Disease Control and Prevention (CDC) was used as a control.

The amplified product was run on 2% agarose gel at 80 V for 40 min. The gel was stained in ethidium bromide, and the bands were visualized under a UV transilluminator.

### Data analysis

Quantitative variables were expressed as medians and interquartile ranges, and categorical variables were expressed as absolute and relative frequencies. The data were analysed with SPSS version 17.0 (IBM Statistical Package for Social Sciences).

## Results

Of the cohort of 48 individuals found to be *Plasmodium* DNA carriers in the first survey, 37 participated in the study and provided blood samples on three different occasions between April 2010 and March 2011. Fifteen (40.5%) of the samples from these 37 asymptomatic carriers had been positive for *P. vivax* DNA in the 2001–2004 survey, 12 (32.4%) for *P. malariae* DNA, 9 (24.3%) for *P. falciparum* DNA, and one (2.7%) for *P. falciparum* and *P. malariae* DNA.

Of the individuals assessed, 25 (67.6%) were males. Median age at the time of the 2010/2011 survey was 24 years, the interquartile range was 18–40 years and the minimum and maximum age were 7 and 62 years, respectively. The most common occupation was farmer (20 individuals or 54.1%) followed by student (9 individuals or 24.3%). The other occupations were land surveyor, shopkeeper, housewife, bank intern, driver (two individuals), bricklayer, and agricultural technician (Table 1).

**Table 1:** Demographic data for 37 residents of a residual malaria transmission area in an Atlantic Forest system

Characteristic	Median (years)	Interquartile range	Frequency, n (%)
Age	24	18–40	–
Gender: male	–	–	25 (67.6)
Occupation: farmer	–	–	20 (54.1)

Six individuals said they had not left the region where they lived during the study. All those who had left the region between the three collections went to nearby municipalities, some of which had residual malaria transmission.

Fourteen individuals reported experiencing fever during the follow-up in at least one of the interviews. In all but one case, in which it subsided after a week, the fever lasted 1–4 days. None of those who presented with fever was tested for malaria by the local health services, and their fevers cleared without any specific treatment. In two individuals the spleen was palpable at the costal margin, in one only at the first collection and in the other at all three collections. Blood smears in both patients were negative for *Plasmodium* spp., and DNA was not amplified in the multiplex PCR. The individual whose spleen was palpable throughout the follow-up (individual no. 34) was seen by the paediatric service at the University Hospital, but the aetiology of the splenomegaly could not be determined. The splenomegaly subsequently regressed and the spleen was no longer palpable.

*Plasmodium malariae* DNA was detected in two individuals. Individual no. 15, who was 18 years old, was positive at the second and third collections, while individual no. 19, who was 24 years old, was positive only at the second collection. Both were male farmers. *Plasmodium malariae* DNA had been detected in these patients’ samples in the first survey as well, although they did not have any symptoms suggestive of malaria in either survey (Table 2). At the first collection, individual no. 15 reported having had a fever that lasted only 1 day three times between 2001 and 2011. Both individuals were treated for malaria after the third collection.

**Table 2:** Data for individuals who were positive by PCR in the 2001–2004 and 2010–2011 surveys in a residual malaria transmission area in an Atlantic Forest system

Individual	Characteristic	2001–2004 survey	2010–2011 survey
15	Age (years)	11	18
	Municipality where individual was living	Santa Maria de Jetibá	Santa Maria de Jetibá
	Symptoms	None	None
	Species identified by PCR	*P. malariae*	*P. malariae*
19	Age (years)	18	24
	Municipality where individual was living	Santa Maria de Jetibá	Santa Maria de Jetibá
	Symptoms	None	None
	Species identified by PCR	*P. malariae*	*P. malariae*

## Discussion

In this study to investigate the persistence of DNA positivity in a cohort of 37 asymptomatic carriers approximately 6 years after an earlier survey, only two patients with a molecular diagnosis consistent with *P. malariae* infection in the first survey still tested positive for *P. malariae* DNA in at least one of the blood samples collected in the present survey, possibly indicating a prolonged, low-parasitaemia infection. This behaviour is expected for a parasite known to circulate unnoticed for long periods in the bloodstream of infected individuals. However, 94.5% of all the asymptomatic carriers assessed were no longer positive for *Plasmodium* DNA regardless of the species originally detected, and positivity for *P. malariae* DNA was observed in only 15.4% (2 out of 13) of the individuals found to be carriers of this species in the first survey. Additionally, none of them developed symptoms compatible with malaria in the period between the first survey and the reassessment.

The role of asymptomatic individuals [12] in the malaria transmission chain in areas where there is a low incidence of the disease has been investigated in various studies in recent years [4, 13–17]. A better understanding of the part played by asymptomatic infections in malaria transmission is essential to implement and improve malaria elimination and eradication programmes. The fact that asymptomatic carriers are not identified by methods currently in regular use is an obstacle to malaria elimination. One of the most important consequences of asymptomatic infection is that those affected do not seek medical assistance or treatment and may therefore help to maintain the malaria cycle.

In an area such as that studied here, where malaria is uncommon and the population would not generally consider it a cause of fever, even people who have been given guidance about the disease do not normally go to medical centres to be tested for malaria infection, unlike in regions of the Amazon where there is a higher incidence of the disease.

In areas of the Atlantic Forest where there is residual malaria and very low endemicity, thick blood smears normally reveal low parasitaemia in symptomatic patients [4]. Most of the infections in these symptomatic individuals are caused by *P. vivax* [4]. As the thick smear test has low sensitivity, a negative result does not necessarily exclude residual parasitaemia. It is thus possible that the absence of definitive clearance of parasitaemia, leading to maintenance of a small number of parasites only detectable by molecular methods, means that patients who are apparently cured in fact act as suppliers of gametocytes, helping to maintain the transmission cycle [18–25].

In a study in the Amazon region, Alves et al. found that even though they were asymptomatic, patients who had exhibited very low parasitaemia, that could only be detected by PCR 2 months before they were recruited to the study to assess their capacity to infect mosquitoes, remained infective. However, they had less infective capacity than the symptomatic patients. They cited unpublished data showing that 40% of the asymptomatic patients were negative by PCR after 2 months of follow-up and suggested that the limited capacity of asymptomatic patients to infect mosquitoes may be made up for by carriage of *Plasmodium* over long periods, potentially maintaining transmission cycles in some regions [26].

An issue that remains to be elucidated is the detection of *P. falciparum* DNA in 10 asymptomatic individuals in the transmission area during the 2001–2004 survey. To date there have been no cases of malaria caused by this species in the region in question, and *P. falciparum* is not considered an aetiologic agent of malaria in Atlantic Forest systems. Recently, Maselli et al. [16] also detected *P. falciparum* DNA in asymptomatic blood donors who had been in the Atlantic Forest system in the state of São Paulo, a state where there have not been any reports of symptomatic cases of this type of malaria among local residents. It is possible that detection of *P. falciparum* could be a result of the molecular technique used. In the 2001–2004 study [4], it was used a multiplex PCR described by Rubio et al. [27]. As the use of multiplex PCR may have affected the specificity of the test, the finding of *P. falciparum* DNA in this region may represent artifacts of the technique, indicating the presence of a species that is not in fact part of the transmission chain [28]. Nevertheless, in a recent study in fragments of Atlantic Forest in the southeast of Brazil, Laporta et al. [29] identified anophelines naturally infected with *P. falciparum* and *P. vivax*, the latter in a smaller proportion of the infected mosquitoes. This was confirmed by DNA sequencing, which identified *P. falciparum* in 76% of the positive specimens, most of which were *Anopheles cruzii*, suggesting that *P. falciparum* circulates actively among *Anopheles* in fragmented areas of the Atlantic Forest in this region of Brazil. In light of these unresolved questions, further studies are needed before the role of *P. falciparum* in the aetiology of residual malaria in Atlantic Forest systems can be determined.

The limitations of this study should also be taken into consideration, as many years had elapsed since the first study, making it difficult to obtain reliable information about the period during which the individuals in the original survey were not followed up. It is not possible to assert without any doubt that the two individuals infected by *P. malariae* in the present survey were not re-infected after the first survey. However, the presence of the same species of parasite in both surveys and the potential for infections of very long duration observed for this species of parasite make it possible that they are the same infections. It should be emphasized that asymptomatic individuals are not treated for malaria in the study area. Consequently, specific treatment does not represent confounding. Information about any symptoms experienced by the participants between blood collections was provided by the participants themselves. At the time of the collections, however, they were all asymptomatic. None of the individuals who reported having had a fever sought medical assistance to be tested for malaria and in all of them the symptoms improved without the use of anti-malarial medication.

## Conclusions

The study’s importance lies in its novelty, as it is the first reassessment of asymptomatic carriers following a prolonged interval of time between surveys. In this cohort, even though asymptomatic carriers were not followed up over a very long period and it was not possible to determine with a reasonable degree of accuracy when DNA tests were no longer likely to be positive, most of the patients who were positive for some of the species in the first survey were no longer positive in this study, apart from two patients who were positive for *P. malariae*. Therefore, although it has been suggested that asymptomatic carriers should be treated [26], treatment in these cases seems clinically irrelevant since the patients with sub-microscopic parasitaemia in the study region, where endemicity is very low, spontaneously ceased to test positive without a specific treatment for malaria. Since most clinical cases of malaria in the region are a consequence of infection by *P. vivax*, the epidemiological impact of a possible long-term carriage of *P. malariae* is limited.
